# Feiyanning Formula Induces Apoptosis of Lung Adenocarcinoma Cells by Activating the Mitochondrial Pathway

**DOI:** 10.3389/fonc.2021.690878

**Published:** 2021-07-02

**Authors:** Li-Min Zhu, Hai-Xia Shi, Masahiro Sugimoto, Kenjiro Bandow, Hiroshi Sakagami, Shigeru Amano, Hai-Bin Deng, Qing-Yu Ye, Yun Gai, Xiao-Li Xin, Zhen-Ye Xu

**Affiliations:** ^1^ Department of Oncology, LongHua Hospital, Shanghai University of Traditional Chinese Medicine, Shanghai, China; ^2^ Department of Traditional Chinese Medicine, Shanghai Ninth People’s Hospital, Shanghai Jiao Tong University School of Medicine, Shanghai, China; ^3^ Research and Development Center for Minimally Invasive Therapies, Institute of Medical Science, Tokyo Medical University, Shinjuku, Japan; ^4^ Division of Biochemistry, Meikai University School of Dentistry, Saitama, Japan; ^5^ Meikai University Research Institute of Odontology (M-RIO), Meikai University School of Dentistry, Saitama, Japan; ^6^ Department of Oncology, The Seventh People’s Hospital, Shanghai University of Traditional Chinese Medicine, Shanghai, China

**Keywords:** FYN, A549 cells, apoptosis, NSCLC, mitochondrial pathway, metabolomics analysis, ATP utilization

## Abstract

Feiyanning formula (FYN) is a traditional Chinese medicine (TCM) prescription used for more than 20 years in the treatment of lung cancer. FYN is composed of *Astragalus membranaceus*, *Polygonatum sibiricum*, *Atractylodes macrocephala*, *Cornus officinalis*, *Paris polyphylla*, and *Polistes olivaceous*, *etc.* All of them have been proved to have anti-tumor effect. In this study, we used the TCM network pharmacological analysis to perform the collection of compound and disease target, the prediction of compound target and biological signal and the Kyoto Encyclopedia of Genes and Genomes (KEGG) pathway enrichment analysis. It was found that the activation of mitochondrial pathway might be the molecular mechanism of the anti-lung cancer effect of FYN. The experimental results showed that FYN had an inhibitory effect on the growth of lung cancer cells in a dose-dependent and time-dependent manner. Moreover, FYN induced G_2_/M cell cycle arrest and apoptotic cell death as early as 6 h after treatment. In addition, FYN significantly induced mitochondrial membrane depolarization and increased calreticulin expression. Metabolomics analysis showed the increase of ATP utilization (assessed by a significant increase of the AMP/ATP and ADP/ATP ratio, necessary for apoptosis induction) and decrease of polyamines (that reflects growth potential). Taken together, our study suggested that FYN induced apoptosis of lung adenocarcinoma cells by promoting metabolism and changing the mitochondrial membrane potential, further supporting the validity of network pharmacological prediction.

## Introduction

Lung cancer has the highest morbidity and mortality worldwide (1). Major lung cancer types include small cell lung cancer (SCLC) and non-small cell lung cancer (NSCLC), with the latter being further divided into adenocarcinoma, squamous cell carcinoma, and large cell carcinoma, accounting for 85 to 90% of all lung cancers (2). Most lung cancers usually produce no specific symptoms at the early stage, and this disease is consequently diagnosed at later stage with poor outcomes. With a 5-year survival rate of only 16.6%, the prognosis for lung cancer is poor. The 5-year survival of lung cancer is less than 4% when distant metastases are discovered at diagnosis (3).

At present, common therapies for lung cancer include surgery, chemotherapy, radiotherapy, targeted therapy, and immunotherapy (4). However, adverse effects such as mucositis, neurotoxicity, and extravasation are often caused by chemotherapy and radiotherapy. Moreover, chemotherapy and targeted therapy easily produce drug resistance. Also, limited benefit of immunotherapy happens in some patients. In China, traditional Chinese medicines (TCMs) are commonly prescribed as an adjuvant therapy to conventional cancer treatments to reduce such adverse reactions to radiotherapy and chemotherapy and improve the efficacy of treatment, thus improving the quality of life of patients and prolonging their survival. Various mechanisms of TCM have been proposed (5) with published evidence that TCM can improve general symptoms and even prolong the survival of cancer patients (6). However, in many instances, TCM is prescribed without objective evidence of mechanism.

Here we sought to better define the activity of Feiyanning formula (FYN), a proprietary prescription developed by Z-YX, a prominent TCM practitioner in Shanghai. The prescription has been used clinically for more than 20 years. Our previous randomized placebo-controlled study showed its benefits in prolonging the patient’s survival and improving the quality of life with limited toxicity and side effects (7). In this study, the Chinese medicine network pharmacology method was adopted to analyze lung cancer as the research object to complete the collection of compounds and disease targets, the prediction of compound targets, the enrichment analysis of biological signals and KEGG pathways to predict the possible role of FYN recipe in lung cancer. To verify the network pharmacological prediction, we performed mitochondrial pathway-related functional verification by observing the effect of FYN on lung cancer cells *in vitro*.

## Methods

### Network Pharmacological Analysis of FYN

#### Collection of Compound Data of 11 Traditional Chinese Medicines

FYN was composed of 11 traditional Chinese medicines. According to the Traditional Chinese Medicine Systems Pharmacology Database and Analysis Platform (TCMSP) (https://tcmspw.com/tcmsp.php), a Bioinformatics Analysis Tool for Molecular mechANism of Traditional Chinese Medicine (BATMAN-TCM) database (http://bionet.ncpsb.org.cn/batman-tcm/) and literature review, their compositional and structural information was collected. In TCMSP data query, the parameters are set as ob ≥30% and dl ≥0.18. In BATMAN data query, the parameter setting score cutoff is 20; adjusted *p*-value is 0.05. The obtained compounds were de-duplicated, and the structural files and SMILES codes of 282 compounds were downloaded from PubChem for the following research.

#### Prediction of Targets of Compounds in 11 Traditional Chinese Medicines

Two methods were used to predict compound targets. First, by Seaware, prediction software of compound activity, 282 compounds were identified. The screening conditions were set for human targets, and potential targets were obtained through calculation and prediction. Second, by SMILES code of compounds, compound targets were predicted from Swiss Target Prediction database (http://www.swisstargetprediction.ch/). The results were obtained by integrating these two methods; the Uniprot database (https://www.uniprot.org/) was used to correct all targets of official gene names.

#### Searching for Anti-Lung Cancer Targets

Anti-lung cancer targets were searched through Comparative Toxicogenomics Database (CTD) (http://ctdbase.org/) with keywords such as “Carcinoma, Non-Small-Cell Lung”, “Carcinoma, Small Cell” and “Small Cell Lung Carcinoma”. Gene names were unified through the Uniprot database. The lung cancer related targets were matched with the potential targets that were calculated in *Prediction of Targets of Compounds in 11 Traditional Chinese Medicines.*, thus the potential action targets of the compound against lung cancer were obtained.

#### Gene Enrichment Analysis

GO (gene ontology) analysis and KEGG analysis were carried out on the targets for the compounds collected through the Database for Annotation, Visualization, and Integrated Discovery (DAVID) (https://david.ncifcrf.gov/, Version 6.8). The biological process (BP), molecular function (MF), and cellular component (CC) are selected for pathway analysis, and the top 20 genes of BP, MF, CC, and KEGG pathways are selected by scoring the *p* values, and the results of pathway enrichment analysis are visualized by R software.

### Experimental Verification

#### Materials and Methods

##### Reagents

FYN prescription powder was provided by the Longhua Hospital Affiliated to Shanghai University of Traditional Chinese Medicine. Dulbecco’s modified Eagle medium (DMEM) was used to dilute the powder to the required concentrations. cisplatin was purchased from Sigma-Aldrich Inc. (St. Louis, MO, USA), and stock solution was prepared with 0.9% sodium chloride.

##### Cell Culture

Lung cancer cell lines, LC-1/sq (squamous cell carcinoma), A549 (adenocarcinoma), WA-hT (small cell carcinoma), and A904 (large cell carcinoma) were purchased from the RIKEN BRC Cell Bank (Tsukuba, Japan). All cell lines were maintained in high glucose DMEM (Thermo Fischer Scientific, Waltham, MA, USA) containing 10% heat-inactivated fetal bovine serum (Fischer Scientific; A3160802), 100 U/ml penicillin G, and 100 μg/ml streptomycin sulfate at 37°C, with a humidified 5% CO_2_ atmosphere. Cells were routinely passaged every 2–3 days, and cells in logarithmic growth phase were used for experiments.

##### Preparation of FYN

FYN is composed of *Astragalus membranaceus*, *Polygonatum sibiricum*, *Atractylodes macrocephala*, *Cornus officinalis*, *Paris polyphylla*, *Polistes olivaceous*, *Salvia chinensis*, *Pseudobulbus cremastrae seu pleiones*, *Corium bufonis*, *Ganoderma lucidum*, and *Epimedii folium*. These herbs were purchased from the Longhua Hospital Affiliated to Shanghai University of TCM (Shanghai, China). The following components were combined as follows: 2,200 ml water was added to the herb mix and soaked for 60 min and filtered with four layers of gauze into 1.8 L filtrate. Then the filtrate was dried directly at −70°C to obtain the lyophilized powder (FYN-M) 41.813 g. Then 500 mg of FYN-M was dissolved in 15 ml of pure water and applied to the activated RP-C_18_ solid phase extraction column (5 g RP-C_18_). The column was washed with 15 ml water to remove carbohydrates and other water-soluble components. Then 15 mg of FYN-M-SPE was eluted with 15 ml chromatographic grade methanol and dissolved with 3 ml methanol/water = 1:1 to make the concentration of 5 mg/ml and dissolved by ultrasonic wave. FYN-M-SPE was further diluted to make 1 mg/ml solution, centrifuged at 15,000 rpm to remove debris. The supernatant was used for the analysis.

#### Cell Proliferation Assays

MTT assays were used to determine the relative viable cell number (8). Briefly, cells were seeded into 96 well plates (4,000 cells/100 μl per well) and allowed to attach before discarding the original medium. Thereafter, 200 μl culture medium (control) or drug-containing medium was added to each well (n = 5 replicates/treatment). Final concentration of FYN was 16, 31, 63, 125, 250, 500, and 1,000 μg/ml. Final concentration of cisplatin was 16, 31, 63, 125, 250, 500, and 1,000 μM. After incubation for 24, 48, and 72 h, the relative viable cell number was determined by MTT method. Cells were incubated for 2 h with 0.2 mg/ml MTT and lysed with dimethyl sulfoxide (DMSO). The absorbance of the cell lysate was then measured at 560 nm, using a microplate reader (Infinite F50R; Tecan, Männedorf, Switzerland). Control cells were treated with the same amounts of DMSO and the cell damage induced by DMSO was subtracted from that induced by test agents. The concentration of compound that reduced the viable cell number by 50% (CC_50_) was determined from the dose–response curve, and the mean value of CC_50_ for each cell type was calculated from triplicate assays.

#### Cell Cycle and Apoptosis Assays

Flow cytometric based analyses were used to measure the effects of FYN on cell cycle and apoptosis using the annexin V-FITC/PI apoptosis detection Kit (Sigma, Aldrich; A9210). Six-well plates were seeded with 5 × 10^5^ A549 cells and cultured overnight. The culture medium was removed and replaced with either: (1) fresh DMEM (blank control) group; (2) 1 mM cisplatin treatment group; or (3) different concentrations of FYN (50, 100, or 200 μg/ml) groups. After 48 h, all cells were collected, washed three times with PBS and then re-suspended with 500 μl binding buffer. Thereafter, 5 μl annexin V-FITC reagent was added and incubated for 15 min at 4°C before adding 5 μl PI solution for 5 min. Cells were then analyzed using flow cytometry (SH800S, Sony Imaging Products & Solutions, Kanagawa, Japan), and cell apoptosis rate was determined using the BD software (9, 10).

#### Metabolomics Analysis

A549 cells were washed twice with 10 ml of 5% mannitol after treating with FYN at different concentrations (10, 30, 60, 100 μg/ml) for 24 h. Aliquots of the cells were trypsinized, and the viable cell number was counted with a hemocytometer after staining with trypan blue. The remaining cells were washed twice with 5 ml of ice-cold 5% D-mannitol and then immersed for 10 min in 1 ml of methanol containing internal standards [25 μM each of methionine sulfone, 2-(*N*-morpholino)-ethane sulfonic acid and D-camphor-10-sulfonic acid]. The methanol extract (supernatant) was collected. The aqueous layer was filtered to remove large molecules by centrifugation through a 5-kDa cutoff filter (Millipore, Billerica, MA) at 9,100 × g for 2.5 h at 4°C. The 320 μl of the filtrate was concentrated by centrifugation and dissolved in 50 μl of Milli-Q water containing reference compounds (200 μM each of 3-aminopyrrolidine and trimesate) immediately before capillary electrophoresis-time-of-flight-mass spectrometry (CE-TOF-MS) analysis. The parameters of the measurement instrument and data processing were described previously. The concentrations of intracellular metabolites were expressed as amol/cell (11).

#### Mitochondrial Membrane Potential

A549 cells were treated with different concentrations of FYN (50, 100 or 200 μg/ml) for 6 h, washed with PBS, and incubated at 37°C for 20 min in 0.5 ml medium containing the JC-1 probe (mitochondrial membrane potential detection kit, Thermo Fisher Scientific, Waltham, MA, USA, T3168). The image was taken with a Zeiss LSM 800 confocal microscope (Carl Zeiss, Oberkochen, Germany) and analyzed with Image J software (https://imagej.nih.gov/ij/).

#### Calreticulin Detection

Cell expression of calreticulin was detected by immunofluorescence staining and flow cytometry. A549 cells were treated with different concentrations of FYN (50, 100 or 200 μg/ml) or cisplatin (1 mM). After 4 h of incubation, the cells were collected and pelleted by centrifugation at 1,200 rpm for 5 min, resuspended with buffer containing Dylight 488 conjugated anti-calreticulin antibody (Enzo Life Sciences, Lausen, Switzerland; ADI-SPA-601-488-D), and incubated for 30 min at 4°C in the dark. Thereafter, cells were resuspended in 500 μl buffer and immediately analyzed by flow cytometer.

#### Statistics

SPSS 22.0 software (IBM, Armonk, NY, USA) was used for statistical analyses, and Prism 6.0 software (GraphPad Software, San Diego, CA, USA) was used for preparing plots. Each experiment was independently repeated three times with data expressed as Mean ± SD. Univariate analysis of variance was used for comparison among groups, and the LSD-t test was used for pairwise comparison within groups. Differences of *p <*0.05 were considered statistically significant.

## Results

### Results of Pharmacological Analysis of FYN Network

#### Collection of Target Points

A total of 1,701 potential targets of TCM components were obtained after the prediction and screening by Seaware reverse target searching and Swiss Target Prediction database. Six hundred forty-six genes related to lung cancer were collected in CTD database. One hundred twenty-four common targets were obtained by intersection of compound targets and human lung adenocarcinoma targets. These 124 common targets are considered as potential targets for the treatment of lung cancer.

#### Network Construction and Analysis

**Figure 1:**
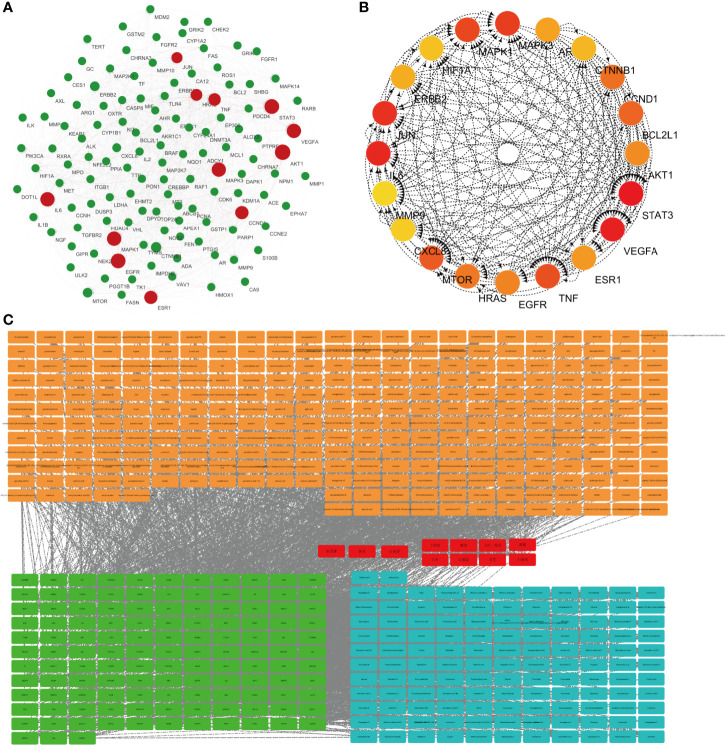
Network pharmacological research of anti-lung cancer target of FYN. **(A)** PPI map of anti-lung cancer related targets of traditional Chinese medicines. **(B)** Top 20 network diagram of key targets. **(C)** Network diagram of traditional Chinese medicine-compound-target (Orange: compounds found in 11 Chinese herbs; Red: 11 Chinese herbs; Green: anti-lung cancer targets of this formula; Blue: compounds corresponding to anti-lung cancer targets).

Protein–protein interaction (PPI) graph is obtained from String database, which contains 124 nodes and 1,485 edges. Using Network Analyzer to analyze the node degree and related parameters, the average node degree is 24, and the average local clustering coefficient is 0.656, as shown in **Figure 1A**.

Traditional Chinese medicine-component-target network diagram is constructed by Cytoscape, as shown in **Figure 1B**. The network includes 540 nodes and 2,751 edges. Among them, nodes represent compounds or targets corresponding to traditional Chinese medicine, while edges indicate the interactions between traditional Chinese medicine and compounds or between compounds and targets. It can be seen from the network diagram that there are phenomena that multiple compounds correspond to one target or one traditional Chinese medicine corresponds to multiple compounds, and there are also situations that multiple targets correspond to one compound or one compound corresponds to multiple traditional Chinese medicines. It indicates that there are pharmacological similarities among several compounds, and it is possible that a single compound can produce anti-lung cancer therapeutic effect through multiple targets. At the same time, side reaction is essential for every traditional Chinese medicine in the anti-lung cancer effect of this prescription.

The CytoHubba module in Cytoscape was used to obtain the top 20 network diagrams of the core target point. The nodes change from orange to red, and the degree value gradually increases from small to large. The importance of the target point was judged according to the degree of value, as shown in **Figure 1C**. The information of top 20 targets was shown in **Table 1**. It can be seen from the network diagram that STAT3, VEGFA, IL6, JUN, and MAPK3 may be the five key targets of the eleven traditional Chinese medicines against lung cancer. Eleven of these targets were associated with apoptosis in lung cancer cells; the result was shown in **Table 2**.

**Table 1:** Degree information of top 20 targets.

Sort	Target spot	Scoring value
1	STAT3	9.00E+18
2	VEGFA	9.00E+18
3	IL6	9.00E+18
4	JUN	9.00E+18
5	MAPK3	9.00E+18
6	MAPK1	9.00E+18
7	TNF	9.00E+18
8	MTOR	8.98E+18
9	BCL2L1	8.98E+18
10	CCND1	8.63E+18
11	HRAS	8.62E+18
12	EGFR	8.59E+18
13	AKT1	8.49E+18
14	ESR1	8.25E+18
15	AR	7.08E+18
16	ERBB2	6.85E+18
17	CTNNB1	6.50E+18
18	HIF1A	5.60E+18
19	CXCL8	5.53E+18
20	MMP9	5.53E+18

**Table 2:** Apoptosis-related targets in lung cancer cells.

Sort	Target spot	Scoring value
1	STAT3	9.00E+18
2	JUN	9.00E+18
3	TNF	9.00E+18
4	MTOR	8.98E+18
5	BCL2L1	8.98E+18
6	CCND1	8.63E+18
7	HRAS	8.62E+18
8	EGFR	8.59E+18
9	AKT1	8.49E+18
10	HIF1A	5.60E+18
11	MMP9	5.53E+18

#### Target-Pathway Network Analysis Diagram

**Figure 2:**
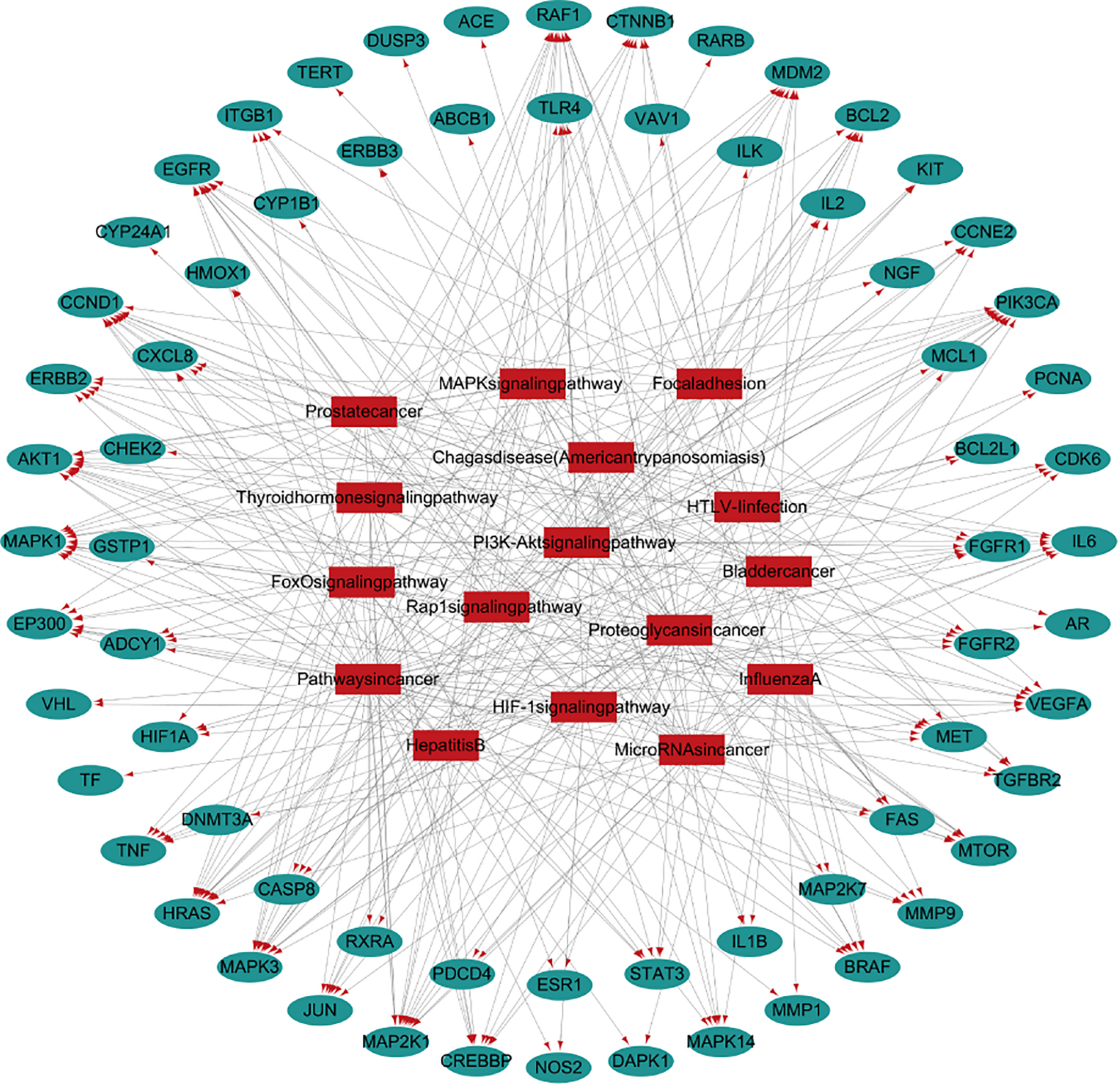
Target-pathway diagram of FYN.

The top 20 pathways were analyzed and the target-pathway analysis diagram was obtained (**Figure 2**). It can be seen from the network diagram that 16 of the top 20 pathways are enriched in the anti-lung cancer genes. Among the 124 potential targets against lung cancer of 11 traditional Chinese medicines, 68 targets were enriched in related pathways.

#### Gene Enrichment Analysis

**Figure 3:**
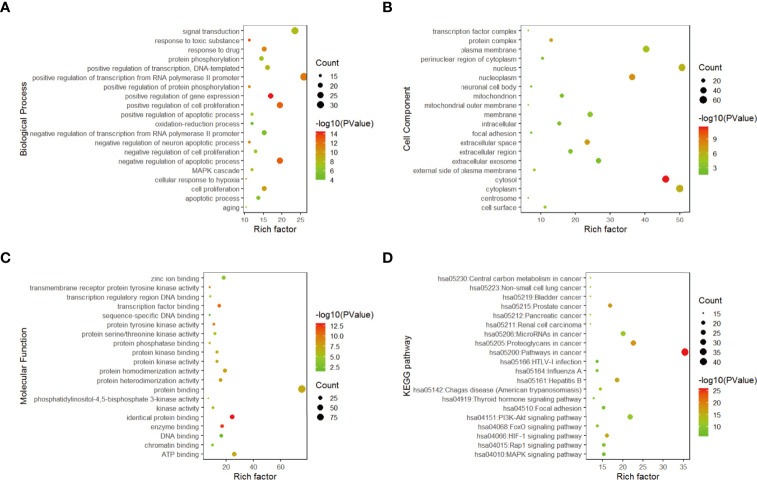
Gene enrichment analysis. **(A)** Top 20 biological process diagram of targets of 11 traditional Chinese medicines. **(B)** Top 20 cell composition diagrame of targets of 11 traditional Chinese medicines. **(C)** Top20 molecular function diagram of **(D)** enrichment analysis of top 20 KEGG pathways.

The functional annotation and pathway enrichment analysis of genes corresponding to 282 compounds were carried out by DAVID platform, and the data of top 20 targets were analyzed according to *p* value and visualized by R software. The results are shown in **Figure 3**. The larger the dots in the figures, the greater the number of enriched genes, and the color of the dots corresponds to *p* value.

From the enrichment results, the biological processes of 282 compounds mainly include: positive regulation of RNA polymerase II promoter transcription, negative regulation of apoptosis process, positive regulation of cell proliferation, positive regulation of gene expression and drug reaction (**Figure 3A**); cell composition mainly includes cell nucleus, cytoplasm, nucleoplasm, and extracellular space (**Figure 3B**). Molecular functions mainly involve protein binding, ATP binding, identical protein binding, enzyme binding, and transcription factor binding (**Figure 3C**).

According to the results of DAVID pathway enrichment analysis, the pathway of top 20 was visualized by R software according to *p* value. The main pathways enriched included cancer pathway, cancer proteoglycan, hepatitis B, prostate cancer, and HIF-1 signaling pathway (**Figure 3D** and **Table 3**).

**Table 3:** KEGG analysis of top 20 pathways.

Access	Number of genes	*p-*Value
hsa05200: Pathways in cancer	44	1.27E-26
hsa05205: Proteoglycans in cancer	28	8.45E-19
hsa04151: PI3K–Akt signaling pathway	27	6.45E-12
hsa05206: MicroRNAs in cancer	25	4.93E-12
hsa05161: Hepatitis B	23	1.71E-16
hsa05215: Prostate cancer	21	1.12E-18
hsa04066: HIF-1 signaling pathway	20	1.42E-16
hsa04510: Focal adhesion	19	1.69E-09
hsa04015: Rap1 signaling pathway	19	2.31E-09
hsa04010: MAPK signaling pathway	19	4.40E-08
hsa05142: Chagas disease (American trypanosomiasis)	18	1.92E-13
hsa04068: FoxO signaling pathway	17	1.45E-10
hsa05164: Influenza A	17	7.17E-09
hsa05166: HTLV-I infection	17	1.39E-06
hsa04919: Thyroid hormone signaling pathway	16	1.61E-10
hsa05219: Bladder cancer	15	4.30E-16
hsa05223: Non-small cell lung cancer	15	6.01E-14
hsa05230: Central carbon metabolism in cancer	15	4.48E-13
hsa05212: Pancreatic cancer	15	5.63E-13
hsa05211: Renal cell carcinoma	15	7.06E-13

#### Pathway Analysis

In recent years, the clinical practice of traditional Chinese medicine has become one of the new approaches for the treatment of cancer (12). We found that the mitochondrial apoptosis pathway is closely related to tumor development. This pathway plays a key role in the process of apoptosis, and the release of cytochrome c (cyt c) is the key link in the mitochondrial apoptosis pathway (13).

According to literature research and pathway map **Figure S1**, it can be found that Bcl-2, an anti-apoptotic protein, forms heterodimer with Bax, and Bak in the mitochondrial apoptotic pathway prevents the oligomerization of Bax and Bak, reduces the permeability of mitochondrial membrane, and leads to the retention of pro-apoptotic factor cyt c in mitochondria, thus reducing the pro-apoptotic effect. After therapy, the ratio of Bax, Bak, and Bcl-2 is unbalanced, or the conformational change and oligomerization of Bax and Bak occur, which caused cyt c to leak into the cytoplasm from mitochondria and then combine with Apaf-1 to form a complex to shear and activate caspase-9, resulting in the activation of downstream caspase-3, thus inducing apoptosis. Therefore, activation of the mitochondrial pathway may be the molecular mechanism of the anti-lung cancer effect of TCM (14).

#### Identification of Major Component of FYN

**Figure 4:**
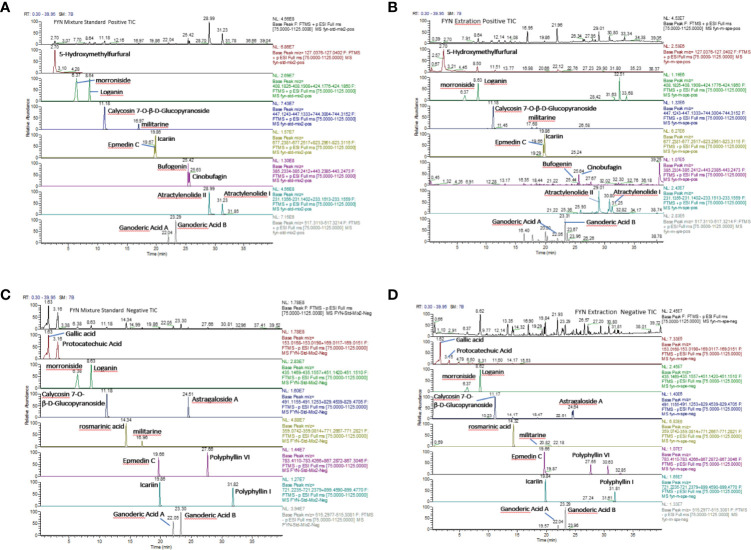
Analysis of FYN by high resolution mass spectrometry. **(A)** Positive total-ion chromatograms of standard by Waters Acquity UPLC-Thermo Q/Exactive HRMS with the mixture standard solution. **(B)** Positive total-ion chromatograms of standard by Waters Acquity UPLC-Thermo Q/Exactive HRMS with FYN extract solution. **(C)** Negative total-ion chromatograms of standard by Waters Acquity UPLC-Thermo Q/Exactive HRMS with the mixture standard solution. **(D)** Negative total-ion chromatograms of standard by Waters Acquity UPLC-Thermo Q/Exactive HRMS with the FYN extract solution.

FYN was analyzed by high resolution mass spectrometry with cationic (A, B) and anionic (C, D) total-ion exchange chromatograms (UPLC- Thermo Q) with standard (**Figure 4**). The most abundant compound identified by cationic ion exchange chromatograms was atractylenolide II, followed by atractylenolide I, bufogenin, calycosin 7-O-*β*-D-glucopyranoside, loganin, morronside, militarine, and epmedin (A, B). The most abundant compound identified by anionic ion exchange chromatograms was gallic acid, component unit of tannin, followed by protocatechuic acid, rosmarinic acid, polyphyllin VI, and ganoderic acid B (C, D).

### Experimental Results

#### FYN Inhibits Human Lung Cancer Cell Proliferation

**Figure 5:**
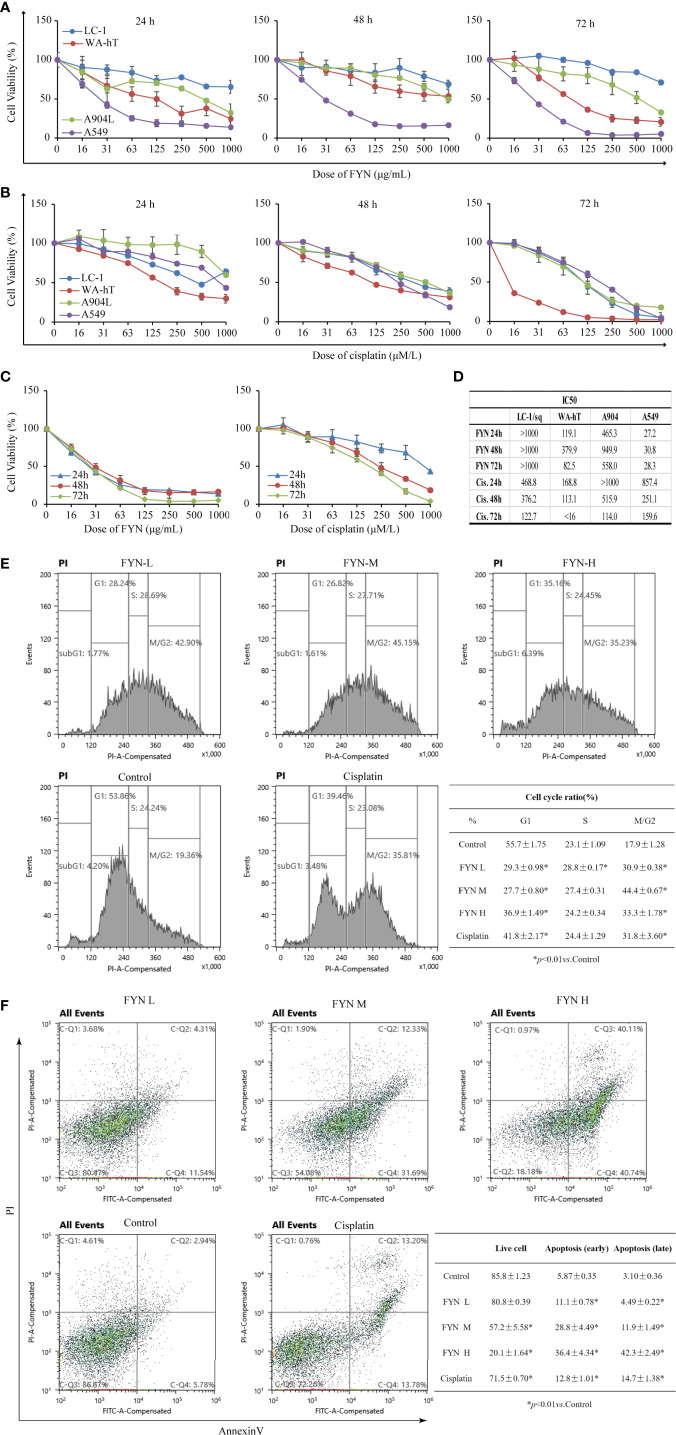
FYN inhibits the proliferation of human lung cancer cells and induces cell cycle arrest and apoptosis. **(A)** Time and dose responses of four lung cancer cell lines to FYN (μg/ml). **(B)** Time and dose responses of the indicated lung cancer cell lines to cisplatin (μM). **(C)** Comparison of FYN *versus* cisplatin effects on A549 cells. **(D)** The IC50 values at each time point of FYN *versus* cisplatin. **(E)** The cell cycle analysis of A549 lung cancer cells after FYN treatment. Flow cytometric profiles of PI staining in A549 cells after treatments without (control) or with FYN, cisplatin. The percentages of cells in the G_1_, S, and G_2_/M phases displayed in the flow cytometric histogram represent one of the three repeated experiments. These data are statistically processed and shown in a table. **(F)** FYN induces apoptosis in A549 lung cancer cells. The cells treated without (control) or with FYN, or cisplatin and stained by FITC-conjugated Annexin V and PI. Then, the cells were analyzed by dual parameter flow cytometry. The percentages of quadrant fractions displayed in the flow cytometric plots represent one of the three repeated experiments. These data are statistically processed and shown in a table.

The analysis of FYN by high resolution mass spectrometry was shown in **Figure 4**. The growth inhibitory effects of FYN were evaluated against four lung cancer cell lines representing the major lung cancer types/subtypes. Analyses using MTT assays showed that the growth of the LC-1/sq squamous cell line, the A549 adenocarcinoma line, WA-hT small cell carcinoma line, and A904 large cell carcinoma line were all significantly reduced after FYN treatment compared to the control group (*p* < 0.01; **Figure 5A**). The inhibitory effects were dose- and time-dependent with maximal inhibition at 72 h. Among the cell lines, FYN showed the strongest inhibitory effect against A549 cells and to a lesser extent in WA-hT cells, and these effects were clearly evident in the first 24 h (**Figure 5A**). In comparison, cisplatin effects were also dose- and time-dependent for all cell lines with WA-hT being the most sensitive (**Figure 5B**). Further research was carried out to compare the cytotoxicity of FYN and cisplatin against A549 cells (**Figure 5C**). The IC50 values at each time point are shown in **Figure 5D**. Since A549 was much more sensitive to FYN than cisplatin, A549 cells were selected to further study the mechanism of action of FYN.

#### Effect of FYN on Apoptosis of A549 Cells

The rapid reduction in cell proliferation observed after 24 h treatment of A549 cells with FYN suggested the occurrence of cell cycle arrest and other types of cell death might occur. Therefore, to determine if FYN induced cell cycle arrest and apoptosis, A549 cells were treated with different concentrations of FYN (25, 50, 100 μg/ml) for 48 h and flow cytometric analyses were performed to measure the cell cycle distribution. Cisplatin (1 mM) was used as a positive control. Compared with control cells, FYN treatment decreased the number of cells in the G1 phase while increasing cells in the G_2/_M phase (*p* < 0.01; **Figure 5E**), suggesting that FYN causes G_2_ cell cycle arrest, similar to the effects of cisplatin. We next investigated the effects of FYN on cell apoptosis. The 24 h FYN treatment of A549 cells promoted both early apoptotic cells (Annexin V+, PI−) and late apoptotic cells (Annexin V+, PI+) in a dose-dependent manner (**Figure 5F**). Collectively, these results indicate that FYN rapidly induces cell cycle arrest and induces apoptosis in A549 lung cancer cells.

#### Metabolomics Analyses of A549 Cells Treated With FYN

**Figure 6:**
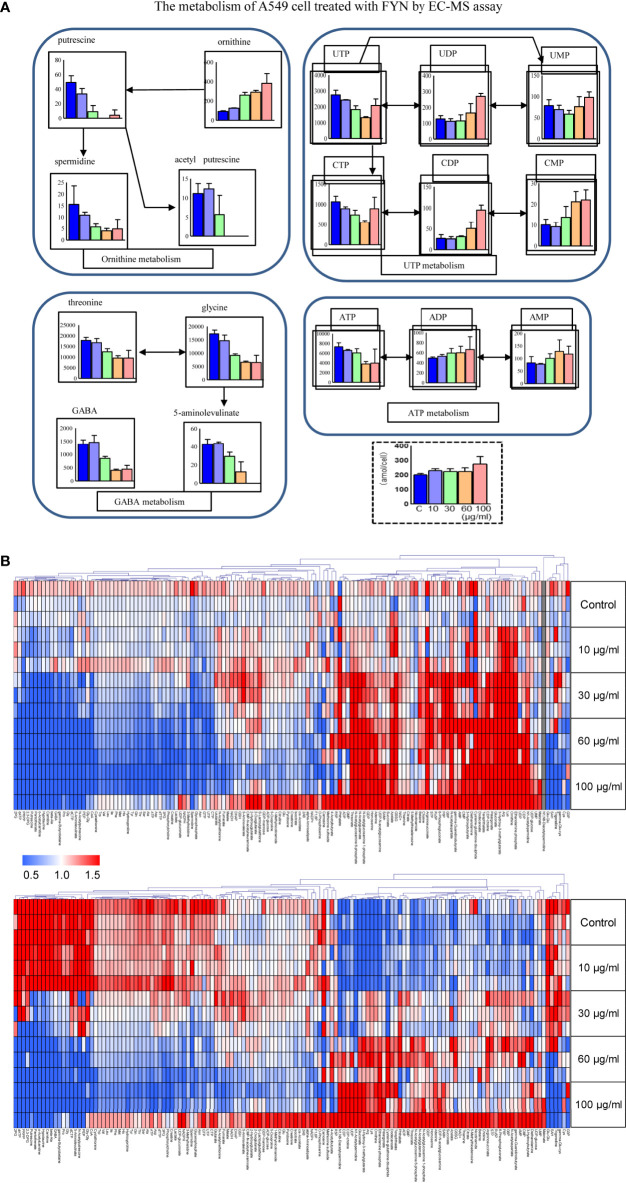
Metabolomics analyses of A549 cells treated with FYN. **(A)** Changes in common cellular metabolites in A549 cells were measured using CE-MS assay after the indicated treatments. A549 cells were treated with different concentrations of FYN (10, 30, 60, 100 μg/ml) for 24 h, and CE-MS was performed to measure cell metabolites (amol/cell). **(B)** Metabolome heat map. Blue, white, and red indicate the fold changes of 0, 1, and 2, respectively, compared to the average of control samples. For undetectable substances in the control group, the half value of the minimum concentration of all samples was used.

Metabolomics was used to determine the changes in the intracellular concentration of metabolites in A549 cells after treatment with FYN. One hundred sixty three metabolites were detected (**Table S1**). **Figure 6B** showed the heat map of metabolites upregulated (red color) or downregulated (blue color), normalized by mean of control samples or mean of total samples. First, there was a significant decrease in putrescine and spermidine in the polyamine pathway (**Figure 6A**). This may reflect the growth inhibition accompanied with apoptosis. ATP cellular content also decreased with accompanying increases in AMP and ADP, suggesting the increased utilization of ATP. Similarly, UTP levels were also decreased with accompanying increases in UMP and UDP, suggesting the increased utilization of UTP. The increased utilization of ATP and UTP, donors of cellular energy, may relate to the stimulated apoptosis induction (**Figure 6A**).

#### Effect of FYN on Mitochondrial Membrane Potential in A549 Cells

**Figure 7:**
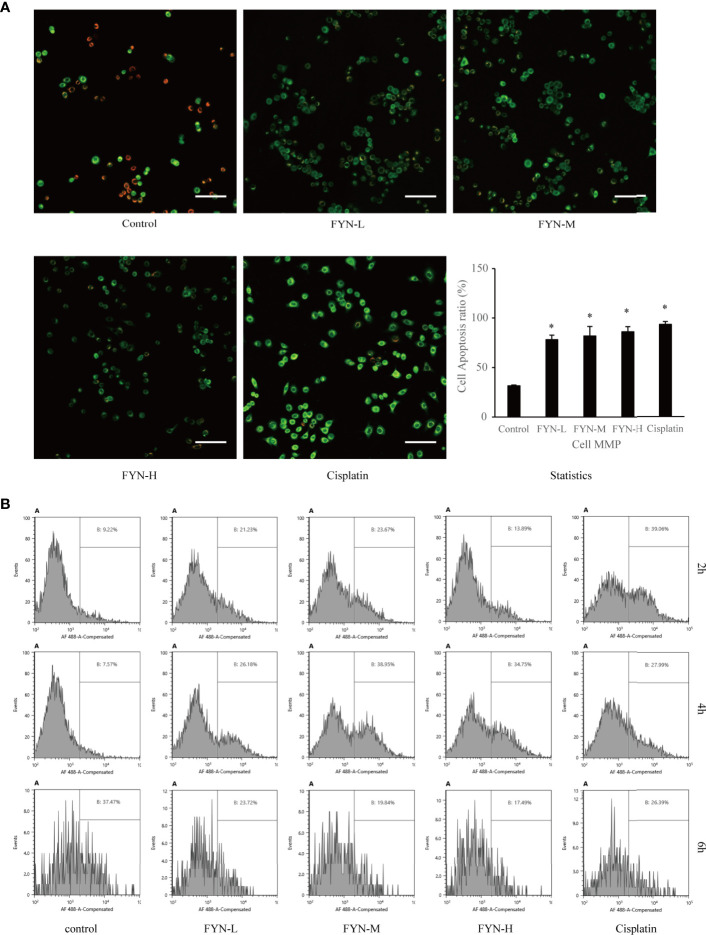
Mitochondrial membrane potential and calreticulin surface expression of A549 cells treated with FYN. **(A)** FYN induces mitochondrial depolarization in A549 cells. A549 cells were treated with different concentrations of FYN for 6 h, and MMP was assessed using JC-1 staining (scale bar: 100 μm). *Significant difference from the value of control group (p<0.05). **(B)** Surface translocation of calreticulin in A549 cells treated with FYN. Flow cytometric profiles of calreticulin staining in A549 cells after the indicated treatments with control, FYN, or cisplatin.

Mitochondrial membrane potential is known to break down in apoptotic or metabolically stressed cells. In view of the fact that FYN can induce rapid apoptosis and also disturbs cellular metabolism, we anticipated there would be accompanying changes in mitochondrial membrane potential. FYN treatment of A549 cells induced significant and dose-dependent decreases in membrane mitochondrial potentials, comparable to cisplatin (**Figure 7A**).

#### The Effect of FYN on Calreticulin Surface Expression

Cellular stress can result in the altered distribution of some intracellular proteins including calreticulin to the cell surface (15). To determine if FYN-induced cell injury causes the translocation of calreticulin to the cell surface, we used a flow cytometric assay. As shown in **Figure 7B**, the surface expression of calreticulin in A549 cells was significantly higher than the untreated control group after FYN treatment, albeit less prominent than after cisplatin treatment (**Figure 7B**).

## Discussion

Lung cancer is the fastest growing malignant tumor in China in terms of morbidity and mortality, which seriously threatens the life of patients (16). Toxicity-based side effects of Western medicines are very common, and drug resistance often develops during repeated courses of treatment. In recent years, targeted therapies and immunotherapy have emerged, but drug resistance still occurs, and the high costs limit availability to many patients. These factors limit the application of cutting-edge Western medicines, particularly in developing countries. The increasing prosperity of China allows more treatment options, but nonetheless, the use of TCM remains high, even in younger generations. Therefore, there is a need to rationally determine the effectiveness and mechanism of action of TCM formulations (17).

FYN has established benefits for the treatment of NSCLC. Previous clinical studies showed that FYN combined with conventional chemotherapy improves reductions in serum tumor biomarkers CA125 and CYFRA21-1, reduces the impact of chemotherapy on the level of peripheral blood lymphocytes, and improves cancer-related fatigue and quality of life scores of patients (18, 19). The clinical effectiveness and disease control rates using FYN were significantly better than those of placebo (20). Our previous basic experiments established that FYN combined with chemotherapeutic drugs induces apoptosis in lung cancer cells (21) and moreover, downregulates the side population (SP) of lung cancers cells, which was reflected by the inhibition of ABCG2 protein expression (22). Furthermore, FYN can inhibit the invasion of A549 lung cancer cells by downregulating Malat1 to inhibit the Wnt/*β*-catenin/EMT signaling axis (23). In this study, we firstly predicted the possible action pathway of FYN through network pharmacology of traditional Chinese medicine, and then demonstrated the direct anticancer effect and its pathway of FYN through *in vitro* experiments. We now build on this foundation to provide a more detailed assessment of the anti-cancer effects of FYN.

We first completed the compound collection involved in FYN, disease target collection, compound target prediction, and KEGG pathway enrichment analysis. It was found that the main targets of FYN were related to the apoptosis of lung cancer cells (24–34) and predicted that the inhibition of FYN on lung cancer growth may be related to the mitochondrial pathway, and then performed *in vitro* experiments. The result showed that in the four representative subtypes of lung cancer, the A549 adenocarcinoma cells were most sensitive to inhibition of cell proliferation by FYN, while FYN inhibited the growth of the WA-hT small cell carcinoma to a lesser extent. Indeed, cell proliferation assays indicated reductions in cell number, possibly by apoptosis, known as physiological process of autonomous cell death. Subsequently, we confirmed that FYN induces cell cycle arrest in the G_2_/M phase resulting in apoptosis. Apoptotic changes were evident as early as 6 h after treatment and notably, the rates of apoptosis at 48 h were similar for 200 μg/ml dose of FYN and the established cytotoxic agent cisplatin. Thus, FYN alone can induce cell cycle arrest and apoptosis in lung cancer cells.

The mitochondria act as the “power house” of cells because of their role in producing energy through aerobic respiration (35). Cellular apoptosis can proceed through the extrinsic and intrinsic pathways, the latter often called the mitochondrial apoptotic pathway (36). When cells are stimulated by DNA damage and/or ATP depletion, mitochondrial membrane swelling and increased permeability can occur to cause the irreversible activation of apoptosis (37). Here mitochondrial stress, defined as the loss of mitochondrial membrane potential, is a key event determining the fate of cells (38). We show here that one of the actions of FYN was the depolarization of mitochondrial membrane potential. Mitochondrial stress signals to activate calcineurin, and many Ca^2+^ response factors, including RYR1, calreticulin, and calcium chelating, are upregulated, as shown in our study (39). Thus, the induction of apoptosis in A549 cells which accompanies the inhibition of cell proliferation by FYN involves the mitochondrial pathway. Accompanying metabonomic profiling of these cells also provided evidence of widespread disturbances in metabolism. FYN decreased the polyamines such as putrescine and spermidine, which is related to growth stimulation (40), accompanied by apoptosis induction. Since apoptosis is an energy-dependent process, an increase in ATP may stimulate the execution of apoptosis (41). This point was further potentiated by our finding that FYN increased the ATP utilization, which is necessary for apoptosis induction.

Finally, we explored the effect of FYN on the translocation of calreticulin to the cell surface. Calreticulin is a calcium binding protein in the endoplasmic reticulum which affects a variety of vital homeostatic processes and plays important roles in cell proliferation, differentiation, and apoptosis (42). Calreticulin is mutated or downregulated in a variety of tumors, and the loss of its function is closely associated with the progression of tumors and the poor treatment prognosis (43). Calreticulin, also known as angiostatin, can inhibit the formation of microvessels and inhibit the growth of tumors. Cellular stress conditions including apoptosis can result in the altered distribution of calreticulin to the cell surface (44). This has also been proposed to facilitate the clearance of dead and dying tumor cells by phagocytes (45). We found that the localization of calreticulin on the cell surface significantly increased, suggesting that calreticulin exposure through FYN-mediated apoptosis may contribute to its anti-cancer properties. However, to determine whether FYN-induced cell injury can prime the lung cancer cells’ removal or can inhibit the blood vessel growth requires further investigation.

In conclusion, we found that FYN can directly induce apoptosis in human lung cancer cells by promoting energy metabolism and change in mitochondrial membrane potential changes. This study provides a rational basis for further investigations on the use of FYN for the treatment of non-small cell lung cancer and other cancers. This finding further supports the validity of network pharmacological prediction.

## Data Availability Statement

The original contributions presented in the study are included in the article/**Supplementary Material**. Further inquiries can be directed to the corresponding authors.

## Author Contributions

L-MZ was responsible for the design and drafting of the article. L-MZ and H-XS acquired, analyzed, and interpreted the data. H-BD provided FYN lyophilized powder. L-MZ, H-XS, MS, KB, HS, and SA participated in data analysis and paper revision of cell experiments. Q-YY, YG, and X-LX were involved in checking the details of the paper format. H-XS and Z-YX gave the final approval and overall responsibility for the published work. All authors contributed to the article and approved the submitted version.

## Funding

The research was funded by the Shanghai Science and Technology Commission Development Foundation, award number is 16ZR1437500; the Shanghai Health and Family Planning Commission of Medical Science and Technology Innovation Project, award number is ZYKC201701009; and The project of “the Sixth Batch of Inheritance of Academic Experience of National Old Chinese Medicine Experts” of the State Administration of Traditional Chinese Medicine, award number is zyyrjf [2017] No. 29.

## Conflict of Interest

The authors declare that the research was conducted in the absence of any commercial or financial relationships that could be construed as a potential conflict of interest.

The reviewer XZ declared a shared affiliation with one of the authors, H-XS, to the handling editor at time of review.
