# Supplementary figures and images for: Feiyanning Formula Induces Apoptosis of Lung Adenocarcinoma Cells by Activating the Mitochondrial Pathway
Source: Front Oncol. 2021 Jul 2;11:690878. doi: 10.3389/fonc.2021.690878 (PMC8284078; doi:10.3389/fonc.2021.690878)

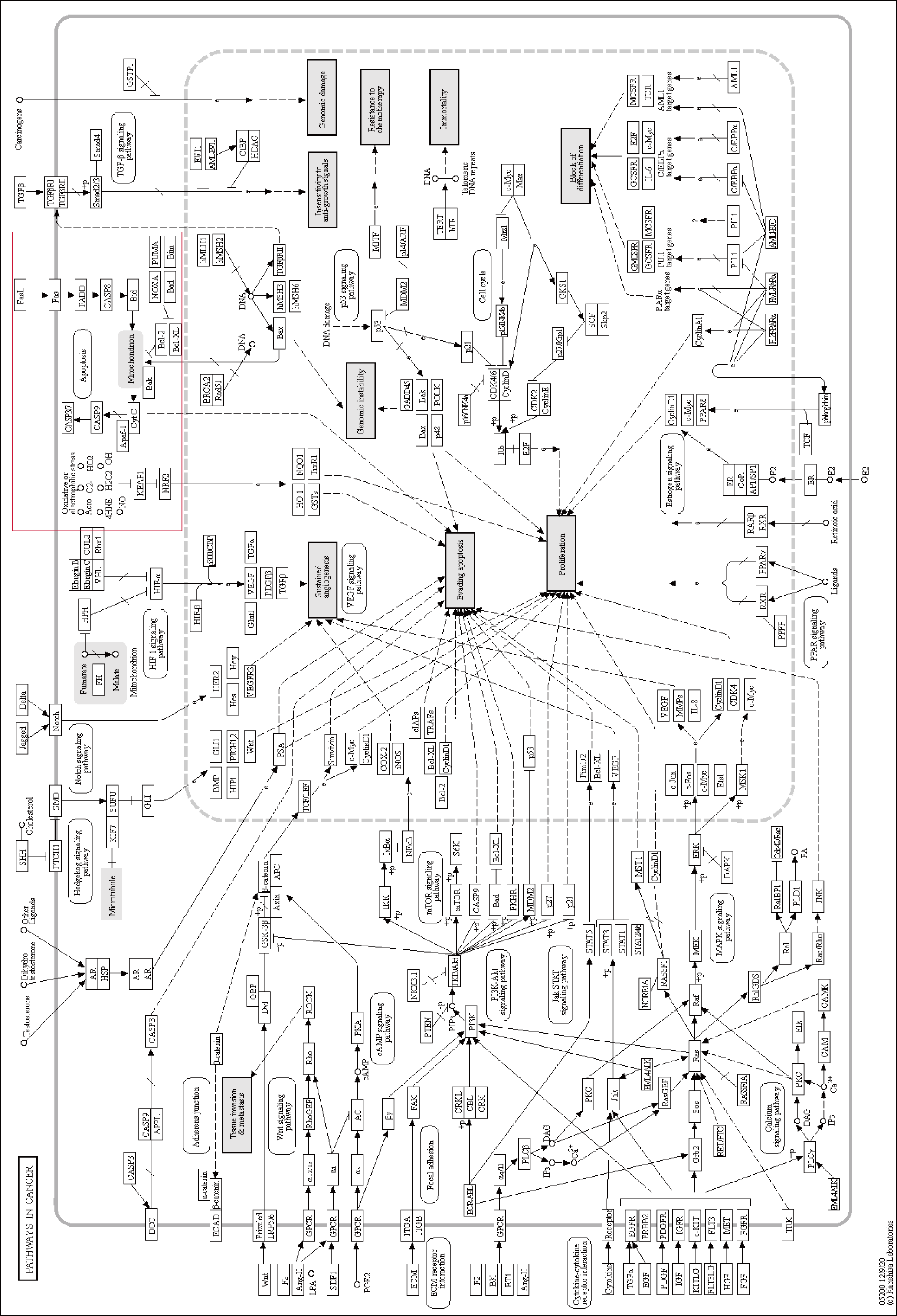

Supplement: Supplementary file 1 [file Image_1.tif]
