# Supplementary material for: Feiyanning Formula Induces Apoptosis of Lung Adenocarcinoma Cells by Activating the Mitochondrial Pathway
Source: Front Oncol. 2021 Jul 2;11:690878. doi: 10.3389/fonc.2021.690878 (PMC8284078; doi:10.3389/fonc.2021.690878)
Supplement: Supplementary file 2 [file Table_1.docx]

**Table S1**

| **KEGG ID** | **Metabolites** | **Ave m/z** | **amol/cell** | | | | | | | | | | | | | | |
| --- | --- | --- | --- | --- | --- | --- | --- | --- | --- | --- | --- | --- | --- | --- | --- | --- | --- |
|  |  |  | **C1** | **C2** | **C3** | **10/1** | **10/2** | **10/3** | **30/1** | **30/2** | **30/3** | **60/1** | **60/2** | **60/3** | **100/1** | **100/2** | **100/3** |
| C00037 | Gly | 76.0403 | 18731 | 15942 | 17089 | 13235 | 13676 | 17107 | 9567 | 9458 | 8497 | 7088 | 6398 | 6285 | 4728 | 5163 | 9593 |
| C00134 | Putrescine(1,4-Butanediamine) | 89.1071 | 59 | 48 | 41 | 28 | 30 | 42 | 10 | 17 | 0 | 0 | 0 | 0 | 0 | 0 | 13 |
| C00213 | Sarcosine | 90.0539 | 32 | 23 | 18 | 26 | 22 | 0 | 21 | 0 | 19 | 0 | 0 | 0 | 0 | 0 | 0 |
| C00099 | beta-Ala | 90.0545 | 6366 | 5469 | 5174 | 5160 | 5067 | 6329 | 2513 | 2490 | 2030 | 956 | 813 | 723 | 468 | 502 | 930 |
| C00041 | Ala | 90.0545 | 5803 | 4890 | 4813 | 4369 | 4186 | 5253 | 3797 | 3474 | 2993 | 3066 | 2659 | 2304 | 1929 | 2161 | 4244 |
| C02356 | 2AB | 104.0713 | 78 | 66 | 66 | 63 | 64 | 80 | 62 | 62 | 59 | 51 | 44 | 57 | 49 | 50 | 96 |
| C00334 | GABA | 104.0713 | 1576 | 1281 | 1322 | 1271 | 1338 | 1769 | 920 | 864 | 772 | 448 | 400 | 361 | 343 | 383 | 611 |
| C00114 | Choline | 104.1071 | 609 | 489 | 544 | 520 | 563 | 621 | 715 | 705 | 705 | 671 | 700 | 712 | 510 | 642 | 1233 |
| C00065 | Ser | 106.0487 | 8578 | 7621 | 7721 | 7365 | 7197 | 8213 | 6535 | 6069 | 5452 | 5332 | 4863 | 4570 | 3923 | 4320 | 7374 |
| C06772 | Diethanolamine | 106.0853 | 400 | 208 | 124 | 134 | 320 | 163 | 555 | 164 | 311 | 1314 | 217 | 475 | 714 | 357 | 1233 |
| C00519 | Hypotaurine | 110.0269 | 4569 | 3930 | 4075 | 3513 | 3377 | 4078 | 1023 | 1157 | 944 | 0 | 0 | 221 | 0 | 0 | 0 |
| C00386 | Carnosine | 114.0595 | 7.4 | 7.7 | 6.5 | 5.8 | 0 | 8.4 | 7.3 | 0 | 7.8 | 0 | 0 | 8.9 | 0 | 0 | 11 |
| C00791 | Creatinine | 114.0667 | 53 | 42 | 45 | 43 | 48 | 49 | 54 | 48 | 44 | 43 | 43 | 42 | 39 | 42 | 67 |
| C00148 | Pro | 116.0713 | 3481 | 2896 | 3123 | 2550 | 2708 | 3391 | 2147 | 2047 | 1903 | 1637 | 1451 | 1268 | 988 | 1067 | 2128 |
| C00183 | Val | 118.0863 | 13235 | 11520 | 11765 | 11773 | 11742 | 13461 | 11509 | 10641 | 9581 | 9562 | 8896 | 7963 | 6821 | 7169 | 12564 |
| C00719 | Betaine | 118.0854 | 267 | 294 | 309 | 307 | 342 | 374 | 395 | 415 | 487 | 352 | 357 | 568 | 240 | 324 | 783 |
| C00431 | 5-Aminovalerate | 118.0852 | 231 | 206 | 215 | 256 | 249 | 296 | 339 | 350 | 257 | 212 | 229 | 185 | 202 | 174 | 272 |
| C00188 | Thr | 120.0653 | 19522 | 16887 | 17118 | 16082 | 15282 | 18972 | 14055 | 12401 | 11115 | 10672 | 9626 | 8438 | 7046 | 7939 | 13707 |
| C00263 | Homoserine | 120.0657 | 21 | 15 | 15 | 20 | 18 | 16 | 23 | 22 | 18 | 24 | 32 | 22 | 27 | 28 | 56 |
| C00097 | Cys | 122.0264 | 51 | 50 | 32 | 35 | 38 | 38 | 48 | 45 | 33 | 25 | 37 | 24 | 24 | 48 | 29 |
| C00153 | Nicotinamide | 123.0555 | 73 | 40 | 46 | 36 | 35 | 38 | 51 | 46 | 38 | 54 | 46 | 47 | 50 | 42 | 98 |
| C02714 | N-Acetylputrescine | 131.1181 | 13 | 8.2 | 13 | 12 | 11 | 14 | 0 | 8.0 | 9.1 | 0 | 0 | 0 | 0 | 0 | 0 |
| C01015 | Hydroxyproline | 132.0659 | 164 | 153 | 151 | 147 | 140 | 180 | 138 | 125 | 116 | 110 | 91 | 99 | 67 | 65 | 155 |
| C00430 | 5-Aminolevulinate | 132.0685 | 49 | 42 | 38 | 43 | 42 | 46 | 34 | 30 | 25 | 19 | 0 | 18 | 0 | 0 | 0 |
| C00300 | Creatine | 132.0766 | 1191 | 1053 | 1096 | 1021 | 1078 | 1267 | 850 | 858 | 714 | 642 | 548 | 479 | 394 | 424 | 807 |
| C00407 | Ile | 132.1024 | 12825 | 11397 | 11833 | 10989 | 11991 | 13365 | 10902 | 10438 | 9568 | 9167 | 8442 | 7848 | 6371 | 6987 | 12223 |
| C00123 | Leu | 132.1024 | 14761 | 12657 | 13075 | 12180 | 13381 | 15082 | 12120 | 11620 | 10609 | 9816 | 9346 | 9045 | 7225 | 7495 | 13842 |
| C00152 | Asn | 133.0592 | 711 | 587 | 601 | 526 | 498 | 592 | 423 | 374 | 336 | 463 | 421 | 359 | 354 | 390 | 639 |
| C02037 | Gly-Gly | 133.0587 | 60 | 57 | 46 | 65 | 56 | 68 | 52 | 57 | 43 | 0 | 0 | 0 | 29 | 0 | 41 |
| C00077 | Ornithine | 133.0970 | 98 | 93 | 84 | 122 | 128 | 127 | 270 | 282 | 227 | 287 | 311 | 273 | 340 | 311 | 496 |
| C00049 | Asp | 134.0440 | 6360 | 5356 | 5504 | 5723 | 5334 | 6496 | 5908 | 5380 | 5248 | 4314 | 3827 | 3271 | 1817 | 2251 | 5140 |
| C00147 | Adenine | 136.0622 | 22 | 19 | 20 | 16 | 18 | 21 | 24 | 27 | 25 | 24 | 34 | 36 | 40 | 38 | 65 |
| C02918 | 1-Methylnicotinamide | 137.0698 | 424 | 387 | 385 | 390 | 398 | 459 | 411 | 411 | 381 | 403 | 379 | 298 | 322 | 351 | 587 |
| C01004 | Trigonelline | 138.0527 | 21 | 15 | 13 | 24 | 15 | 17 | 25 | 24 | 17 | 24 | 0 | 11 | 0 | 0 | 0 |
| C01035 | gamma-Guanidinobutyrate | 146.0911 | 30 | 29 | 32 | 35 | 31 | 32 | 42 | 34 | 39 | 48 | 37 | 32 | 34 | 37 | 50 |
| C01181 | gamma-Butyrobetaine | 146.1161 | 71 | 62 | 69 | 51 | 54 | 63 | 31 | 30 | 28 | 23 | 15 | 18 | 0 | 0 | 24 |
| C00315 | Spermidine | 146.1674 | 25 | 11 | 10 | 9.8 | 12 | 11 | 7.2 | 5.1 | 4.7 | 3.1 | 3.6 | 5.2 | 0 | 6.8 | 7.6 |
| C00064 | Gln | 147.0767 | 53379 | 45887 | 46128 | 45940 | 43589 | 51716 | 39305 | 35857 | 34017 | 30663 | 27933 | 27255 | 21308 | 23504 | 39920 |
| C00047 | Lys | 147.1130 | 2164 | 1730 | 1669 | 2689 | 2791 | 2909 | 6446 | 6288 | 5759 | 7406 | 7828 | 5753 | 6631 | 6500 | 10983 |
| C00025 | Glu | 148.0614 | 68252 | 60852 | 59692 | 60906 | 64518 | 69471 | 72246 | 66969 | 59795 | 60686 | 57625 | 45377 | 43325 | 48842 | 84258 |
| C00073 | Met | 150.0586 | 2775 | 2390 | 2534 | 2423 | 2499 | 2891 | 2302 | 2203 | 1935 | 1866 | 1701 | 1585 | 1358 | 1474 | 2560 |
| C00135 | His | 156.0765 | 2939 | 2531 | 2712 | 2572 | 2601 | 3124 | 2404 | 2226 | 2013 | 2046 | 1785 | 1659 | 1415 | 1509 | 2619 |
| C00956 | alpha-Aminoadipate | 162.0762 | 79 | 56 | 75 | 65 | 93 | 69 | 65 | 88 | 85 | 72 | 48 | 45 | 40 | 44 | 93 |
| C00318 | Carnitine | 162.1105 | 530 | 475 | 464 | 366 | 353 | 446 | 175 | 164 | 148 | 87 | 73 | 74 | 52 | 52 | 107 |
| C02989 | Methionine sulfoxide | 166.0531 | 78 | 90 | 99 | 89 | 96 | 128 | 73 | 96 | 103 | 68 | 87 | 97 | 68 | 84 | 131 |
| C00079 | Phe | 166.0865 | 7767 | 6565 | 6952 | 6534 | 6690 | 7970 | 6195 | 5801 | 5258 | 5091 | 4589 | 4228 | 3614 | 3738 | 6920 |
| C00250 | Pyridoxal | 168.0627 | 20 | 14 | 13 | 0 | 15 | 20 | 15 | 15 | 0 | 0 | 0 | 0 | 0 | 0 | 0 |
| C00314 | Pyridoxine | 170.0811 | 69 | 54 | 62 | 55 | 57 | 68 | 65 | 59 | 50 | 54 | 54 | 52 | 53 | 48 | 86 |
| C01152 | 3-Methylhistidine | 170.0907 | 51 | 47 | 39 | 51 | 44 | 52 | 55 | 40 | 39 | 37 | 38 | 30 | 34 | 29 | 52 |
| C00062 | Arg | 175.1178 | 895 | 688 | 672 | 1096 | 1113 | 1104 | 2742 | 2567 | 2257 | 2877 | 3266 | 2515 | 2976 | 2863 | 4635 |
| C00327 | Citrulline | 176.1031 | 88 | 71 | 70 | 78 | 78 | 86 | 81 | 77 | 64 | 66 | 61 | 60 | 49 | 55 | 107 |
| C00082 | Tyr | 182.0805 | 9103 | 7871 | 7866 | 8103 | 7362 | 9012 | 7426 | 6340 | 5823 | 6048 | 5491 | 4657 | 4044 | 4333 | 7628 |
| C00588 | Phosphorylcholine | 184.0738 | 64443 | 55829 | 55580 | 56879 | 59547 | 68288 | 54547 | 52316 | 44853 | 33070 | 28174 | 22474 | 16665 | 18712 | 40457 |
| C00021 | SAH | 193.0672 | 18 | 11 | 11 | 11 | 13 | 8.8 | 9.3 | 12 | 11 | 15 | 0 | 0 | 0 | 0 | 0 |
| C00019 | SAM+ | 200.0755 | 357 | 251 | 269 | 277 | 267 | 246 | 359 | 324 | 296 | 344 | 401 | 303 | 498 | 450 | 666 |
| C02571 | o-Acetylcarnitine | 204.1209 | 137 | 118 | 102 | 91 | 84 | 96 | 43 | 43 | 32 | 20 | 21 | 18 | 17 | 17 | 16 |
| C00078 | Trp | 205.0971 | 1561 | 1325 | 1391 | 1306 | 1386 | 1606 | 1325 | 1248 | 1107 | 1110 | 1059 | 945 | 850 | 883 | 1477 |
| - | Cysteine-glutathione disulphide | 214.0497 | 19 | 0 | 18 | 42 | 48 | 0 | 31 | 28 | 0 | 101 | 151 | 93 | 168 | 56 | 129 |
| C02291 | Cystathionine | 223.0786 | 225 | 205 | 215 | 180 | 195 | 250 | 185 | 159 | 172 | 166 | 149 | 89 | 93 | 95 | 200 |
| C00669 | gamma-Glu-cys | 251.0657 | 608 | 509 | 570 | 504 | 527 | 694 | 844 | 618 | 678 | 380 | 521 | 354 | 0 | 0 | 262 |
| C00378 | Thiamine | 265.1099 | 601 | 537 | 492 | 506 | 494 | 557 | 625 | 578 | 544 | 623 | 574 | 530 | 540 | 591 | 923 |
| C01425 | Glu-Glu | 277.1022 | 26 | 23 | 22 | 25 | 27 | 24 | 26 | 22 | 21 | 21 | 0 | 0 | 0 | 0 | 0 |
| C00170 | 5-Methylthioadenosine | 298.0953 | 48 | 30 | 33 | 30 | 35 | 31 | 34 | 37 | 27 | 41 | 41 | 24 | 30 | 33 | 71 |
| C00127 | Glutathione(ox) | 307.0839 | 3826 | 1681 | 4126 | 6941 | 7666 | 3050 | 5649 | 4984 | 5685 | 10359 | 13025 | 9134 | 6708 | 4418 | 20645 |
| C00051 | Glutathione(red) | 308.0920 | 116517 | 105624 | 100101 | 104610 | 103885 | 129022 | 148776 | 138358 | 118212 | 108063 | 91638 | 76855 | 74723 | 97259 | 129419 |
| C03451 | S-Lactoylglutathione | 380.1115 | 60 | 51 | 54 | 69 | 59 | 73 | 78 | 74 | 69 | 72 | 58 | 58 | 39 | 62 | 109 |
| C00612 | N1-Acetylspermidine | 188.1743 | 15 | 14 | 15 | 19 | 18 | 17 | 19 | 21 | 19 | 36 | 54 | 35 | 280 | 213 | 170 |
| C00612 | N1-Acetylspermidine | 188.1743 | 6.0 | 5.3 | 5.9 | 7.4 | 6.9 | 6.6 | 7.4 | 8.2 | 7.5 | 14 | 21 | 14 | 110 | 83 | 67 |
| C00022 | Pyruvate | 87.0101 | 955 | 613 | 658 | 636 | 645 | 839 | 781 | 0 | 0 | 0 | 0 | 0 | 0 | 0 | 0 |
| C00186 | Lactate | 89.0257 | 23030 | 16129 | 15761 | 16246 | 15376 | 18526 | 17960 | 16302 | 14867 | 13735 | 12527 | 9259 | 9828 | 10782 | 16732 |
| C00383 | Malonate | 103.0049 | 65 | 48 | 61 | 36 | 48 | 61 | 46 | 64 | 56 | 59 | 51 | 53 | 58 | 85 | 82 |
| C01089 | 3-Hydroxybutyrate | 103.0405 | 107 | 80 | 62 | 103 | 127 | 113 | 132 | 94 | 98 | 169 | 102 | 86 | 136 | 120 | 197 |
| C00122 | Fumarate | 115.0047 | 511 | 397 | 318 | 421 | 425 | 407 | 524 | 461 | 346 | 387 | 346 | 232 | 177 | 217 | 393 |
| C00042 | Succinate | 117.0204 | 192 | 198 | 212 | 245 | 226 | 217 | 240 | 229 | 201 | 235 | 241 | 192 | 260 | 237 | 332 |
| C01879 | 5-Oxoproline | 128.0363 | 2764 | 2408 | 2240 | 2355 | 2369 | 2491 | 2651 | 2499 | 2162 | 2438 | 2314 | 1982 | 2042 | 2015 | 3322 |
| C02129 | 4-Acetylbutyrate | 129.0561 | 41 | 22 | 37 | 0 | 30 | 31 | 31 | 36 | 26 | 34 | 54 | 43 | 0 | 0 | 66 |
| C00489 | Glutarate | 131.0358 | 134 | 118 | 96 | 132 | 107 | 125 | 158 | 118 | 101 | 104 | 74 | 0 | 0 | 72 | 140 |
| C00711 | Malate | 133.0155 | 1461 | 1289 | 1228 | 1420 | 1427 | 1448 | 1585 | 1647 | 1412 | 1385 | 1572 | 930 | 796 | 866 | 1503 |
| C00346 | Ethanolamine phosphate | 140.0131 | 1494 | 1656 | 1544 | 2216 | 2391 | 1922 | 5232 | 5246 | 4999 | 6664 | 7806 | 7695 | 8340 | 8378 | 8303 |
| C06423 | Octanoate | 143.1088 | 52 | 47 | 46 | 29 | 0 | 0 | 0 | 0 | 37 | 0 | 0 | 0 | 0 | 0 | 0 |
| C00026 | 2-Oxoglutarate | 145.0156 | 857 | 780 | 736 | 813 | 846 | 897 | 924 | 1005 | 848 | 992 | 848 | 801 | 587 | 969 | 1296 |
| C02630 | 2-Hydroxyglutarate | 147.0314 | 83 | 67 | 71 | 77 | 82 | 76 | 92 | 98 | 74 | 89 | 90 | 62 | 51 | 82 | 136 |
| C01601 | Pelargonate | 157.1250 | 42 | 32 | 45 | 42 | 40 | 37 | 56 | 56 | 45 | 65 | 58 | 54 | 74 | 83 | 72 |
| C03761 | 3-Hydroxy-3-methylglutarate | 161.0467 | 50 | 39 | 45 | 66 | 60 | 59 | 157 | 155 | 110 | 237 | 208 | 170 | 234 | 270 | 350 |
| C06337 | Terephthalate | 165.0211 | 27 | 23 | 26 | 28 | 24 | 26 | 24 | 33 | 34 | 43 | 40 | 36 | 52 | 38 | 44 |
| C01606 | Phthalate | 165.0203 | 0 | 4.0 | 0 | 3.4 | 0 | 2.5 | 5.5 | 4.9 | 5.6 | 5.9 | 7.4 | 0 | 0 | 5.6 | 14 |
| C00074 | PEP | 166.9771 | 46 | 37 | 49 | 35 | 36 | 53 | 0 | 31 | 37 | 22 | 0 | 0 | 0 | 0 | 0 |
| C00111 | DHAP | 168.9924 | 871 | 908 | 678 | 896 | 878 | 1037 | 1091 | 869 | 985 | 818 | 660 | 685 | 629 | 809 | 1004 |
| C00093 | Glycerophosphate | 171.0076 | 401 | 245 | 243 | 228 | 198 | 238 | 172 | 187 | 152 | 201 | 172 | 112 | 120 | 138 | 222 |
| C00417 | cis-Aconitate | 173.0104 | 107 | 96 | 101 | 98 | 96 | 118 | 102 | 95 | 90 | 88 | 87 | 79 | 83 | 96 | 139 |
| C01042 | N-Acetylaspartate | 174.0422 | 1255 | 1084 | 1110 | 1421 | 1420 | 1471 | 2167 | 2059 | 1857 | 2269 | 2224 | 1739 | 1750 | 1913 | 2988 |
| C01005 | O-Phosphoserine | 184.0031 | 46 | 45 | 67 | 42 | 39 | 53 | 37 | 40 | 0 | 27 | 43 | 36 | 0 | 55 | 61 |
| C00197 | 3PG | 184.9876 | 176 | 164 | 179 | 136 | 165 | 207 | 151 | 153 | 135 | 92 | 73 | 72 | 72 | 61 | 140 |
| C08261 | Azelate | 187.0995 | 12 | 7.5 | 16 | 16 | 17 | 11 | 14 | 29 | 15 | 31 | 23 | 19 | 21 | 22 | 56 |
| C00624 | N-Acetylglutamate | 188.0577 | 82 | 74 | 66 | 80 | 77 | 85 | 130 | 115 | 94 | 138 | 129 | 110 | 113 | 138 | 211 |
| C02712 | N-Acetylmethionine | 190.0554 | 32 | 27 | 24 | 35 | 32 | 33 | 33 | 30 | 26 | 26 | 26 | 21 | 16 | 17 | 29 |
| C00311 | Isocitrate | 191.0216 | 94 | 78 | 76 | 86 | 73 | 108 | 76 | 79 | 67 | 61 | 57 | 71 | 62 | 81 | 157 |
| C00158 | Citrate | 191.0216 | 1682 | 1502 | 1544 | 1616 | 1618 | 1822 | 1761 | 1674 | 1526 | 1663 | 1570 | 1526 | 1636 | 1755 | 2425 |
| C00257 | Gluconate | 195.0524 | 431 | 375 | 356 | 420 | 452 | 461 | 912 | 861 | 772 | 1367 | 1418 | 1136 | 1608 | 1787 | 2403 |
| C02679 | Dodecanoate | 199.1719 | 106 | 99 | 93 | 109 | 109 | 104 | 124 | 124 | 130 | 168 | 188 | 151 | 187 | 139 | 162 |
| C00864 | Pantothenate | 218.1049 | 395 | 374 | 331 | 232 | 238 | 276 | 91 | 86 | 76 | 67 | 51 | 50 | 72 | 55 | 71 |
| C00117 | R5P | 229.0125 | 62 | 44 | 77 | 72 | 48 | 73 | 75 | 66 | 83 | 47 | 82 | 65 | 50 | 44 | 101 |
| C00199 | Ru5P | 229.0131 | 192 | 160 | 150 | 172 | 150 | 205 | 242 | 215 | 210 | 213 | 216 | 193 | 153 | 171 | 254 |
| C00092 | G6P | 259.0241 | 322 | 249 | 194 | 212 | 209 | 265 | 427 | 386 | 385 | 512 | 356 | 288 | 265 | 332 | 512 |
| C00085 | F6P | 259.0245 | 100 | 75 | 53 | 76 | 68 | 93 | 139 | 127 | 112 | 164 | 130 | 106 | 92 | 94 | 189 |
| C01159 | 2,3-DPG | 264.9538 | 57 | 50 | 48 | 39 | 32 | 50 | 32 | 29 | 0 | 0 | 21 | 0 | 0 | 0 | 0 |
| C00345 | 6-Phosphogluconate | 275.0176 | 59 | 54 | 59 | 51 | 47 | 50 | 81 | 73 | 62 | 82 | 65 | 68 | 55 | 77 | 107 |
| C05382 | S7P | 289.0343 | 78 | 63 | 59 | 59 | 64 | 74 | 102 | 71 | 98 | 118 | 101 | 71 | 75 | 64 | 113 |
| C03406 | Argininosuccinate | 289.1171 | 33 | 0 | 18 | 40 | 0 | 35 | 67 | 47 | 45 | 81 | 64 | 60 | 52 | 51 | 70 |
| C00357 | N-Acetylglucosamine 6-phosphate | 300.0504 | 47 | 45 | 48 | 53 | 52 | 59 | 83 | 90 | 71 | 107 | 112 | 101 | 92 | 108 | 204 |
| C04501 | N-Acetylglucosamine 1-phosphate | 300.0506 | 104 | 87 | 90 | 107 | 98 | 99 | 166 | 130 | 128 | 243 | 218 | 174 | 202 | 254 | 447 |
| C00055 | CMP | 322.0467 | 12 | 12 | 7.0 | 12 | 7.9 | 7.9 | 19 | 8.9 | 13 | 20 | 26 | 17 | 19 | 20 | 27 |
| C00105 | UMP | 323.0302 | 91 | 80 | 63 | 58 | 77 | 73 | 61 | 66 | 49 | 74 | 101 | 54 | 87 | 95 | 112 |
| C00354 | F1,6P | 338.9911 | 986 | 1441 | 1362 | 1369 | 1378 | 1406 | 1279 | 1128 | 832 | 813 | 1063 | 707 | 623 | 854 | 1735 |
| C00020 | AMP | 346.0579 | 111 | 72 | 65 | 79 | 75 | 80 | 122 | 93 | 88 | 124 | 176 | 85 | 89 | 113 | 151 |
| C00144 | GMP | 362.0521 | 32 | 32 | 30 | 32 | 24 | 24 | 43 | 31 | 25 | 33 | 45 | 16 | 31 | 28 | 61 |
| C00005 | NADPH | 371.5414 | 252 | 208 | 174 | 176 | 202 | 220 | 206 | 162 | 121 | 93 | 96 | 52 | 85 | 92 | 270 |
| C00010 | CoA | 382.5530 | 41 | 32 | 30 | 30 | 29 | 39 | 14 | 21 | 17 | 15 | 16 | 0 | 0 | 6.6 | 25 |
| C00119 | PRPP | 388.9465 | 114 | 64 | 76 | 67 | 52 | 46 | 38 | 52 | 0 | 0 | 0 | 0 | 0 | 0 | 0 |
| C00112 | CDP | 402.0128 | 23 | 37 | 21 | 31 | 25 | 21 | 33 | 31 | 28 | 35 | 66 | 52 | 81 | 102 | 102 |
| C00015 | UDP | 402.9969 | 137 | 141 | 104 | 127 | 116 | 94 | 141 | 133 | 70 | 124 | 232 | 139 | 250 | 287 | 270 |
| C00008 | ADP | 426.0244 | 506 | 508 | 468 | 568 | 527 | 497 | 671 | 618 | 494 | 587 | 737 | 479 | 480 | 559 | 958 |
| C00035 | GDP | 442.0187 | 81 | 68 | 63 | 77 | 80 | 74 | 90 | 95 | 80 | 104 | 137 | 106 | 108 | 104 | 183 |
| C00458 | dCTP | 465.9841 | 32 | 24 | 24 | 16 | 18 | 25 | 20 | 16 | 15 | 0 | 0 | 0 | 0 | 0 | 0 |
| C00459 | dTTP | 480.9839 | 58 | 50 | 53 | 52 | 50 | 61 | 49 | 42 | 48 | 31 | 31 | 32 | 25 | 30 | 55 |
| C00063 | CTP | 481.9795 | 1219 | 962 | 1005 | 932 | 907 | 841 | 851 | 754 | 618 | 522 | 601 | 549 | 675 | 792 | 1212 |
| C00075 | UTP | 482.9638 | 3082 | 2509 | 2663 | 2424 | 2398 | 2450 | 2005 | 1918 | 1573 | 1261 | 1319 | 1383 | 1716 | 2009 | 2528 |
| C00131 | dATP | 489.9972 | 72 | 46 | 58 | 50 | 61 | 51 | 54 | 48 | 0 | 0 | 0 | 0 | 0 | 0 | 0 |
| C00002 | ATP | 505.9909 | 8248 | 6722 | 6972 | 6772 | 6586 | 6411 | 6789 | 6307 | 5123 | 4222 | 3913 | 3221 | 2012 | 2586 | 7278 |
| C00044 | GTP | 521.9858 | 1297 | 873 | 920 | 820 | 778 | 563 | 831 | 747 | 601 | 470 | 589 | 450 | 421 | 432 | 1218 |
| C00307 | CDP-choline | 547.1237 | 41 | 59 | 43 | 64 | 59 | 47 | 89 | 99 | 74 | 91 | 106 | 94 | 183 | 179 | 173 |
| C00029 | UDP-glucose | 565.0505 | 1140 | 1072 | 908 | 1119 | 1011 | 1179 | 1178 | 1066 | 827 | 960 | 933 | 861 | 1250 | 1436 | 1627 |
| C00167 | UDP-glucuronate | 579.0298 | 1236 | 1104 | 1051 | 1094 | 1035 | 1248 | 1131 | 982 | 712 | 748 | 697 | 545 | 614 | 758 | 1592 |
| C00498 | ADP-glucose | 588.0794 | 38 | 35 | 39 | 38 | 35 | 40 | 41 | 39 | 38 | 33 | 36 | 27 | 23 | 25 | 61 |
| C00096 | GDP-mannose | 604.0711 | 58 | 38 | 36 | 39 | 42 | 46 | 52 | 41 | 42 | 38 | 35 | 34 | 28 | 44 | 56 |
| C00043 | UDP-N-acetylglucosamine | 606.0771 | 1657 | 1391 | 1365 | 1505 | 1424 | 1652 | 1718 | 1495 | 1252 | 1825 | 1776 | 1648 | 2075 | 2610 | 3484 |
| C00128 | CMP-N-acetylneuraminate | 613.1416 | 103 | 90 | 80 | 104 | 96 | 100 | 119 | 110 | 90 | 111 | 85 | 80 | 56 | 81 | 139 |
| C00003 | NAD+ | 662.1052 | 1090 | 963 | 866 | 1033 | 1011 | 1093 | 1263 | 1192 | 1020 | 1223 | 1176 | 966 | 987 | 1044 | 1674 |
| C00004 | NADH | 664.1193 | 158 | 178 | 157 | 144 | 157 | 189 | 224 | 184 | 133 | 154 | 181 | 110 | 100 | 112 | 217 |
| C00006 | NADP+ | 742.0702 | 48 | 53 | 44 | 58 | 49 | 46 | 36 | 42 | 41 | 46 | 30 | 28 | 39 | 38 | 78 |
| C01620 | Threonate | 135.0309 | 684 | 653 | 605 | 749 | 760 | 824 | 1310 | 1331 | 1085 | 1864 | 1658 | 1244 | 1433 | 1440 | 2957 |
| C00661 | G3P | 168.9938 | 178 | 76 | 94 | 85 | 86 | 56 | 104 | 99 | 79 | 0 | 78 | 79 | 143 | 0 | 0 |
| C00631 | 2PG | 184.9873 | 28 | 14 | 27 | 24 | 28 | 25 | 21 | 0 | 16 | 0 | 0 | 0 | 0 | 0 | 0 |
